# Nogo-66 Promotes the Differentiation of Neural Progenitors into Astroglial Lineage Cells through mTOR-STAT3 Pathway

**DOI:** 10.1371/journal.pone.0001856

**Published:** 2008-03-26

**Authors:** Bin Wang, Zhifeng Xiao, Bing Chen, Jin Han, Yuan Gao, Jing Zhang, Wenxue Zhao, Xia Wang, Jianwu Dai

**Affiliations:** Key Laboratory of Molecular Developmental Biology, Institute of Genetics and Developmental Biology, Chinese Academy of Sciences, Beijing, China; National Institutes of Health, United States of America

## Abstract

**Background:**

Neural stem/progenitor cells (NPCs) can differentiate into neurons, astrocytes and oligodendrocytes. NPCs are considered valuable for the cell therapy of injuries in the central nervous system (CNS). However, when NPCs are transplanted into the adult mammalian spinal cord, they mostly differentiate into glial lineage. The same results have been observed for endogenous NPCs during spinal cord injury. However, little is known about the mechanism of such fate decision of NPCs.

**Methodology/Principal Findings:**

In the present study, we have found that myelin protein and Nogo-66 promoted the differentiation of NPCs into glial lineage. NgR and mTOR-Stat3 pathway were involved in this process. Releasing NgR from cell membranes or blocking mTOR-STAT3 could rescue the enhanced glial differentiation by Nogo-66.

**Conclusions/Significance:**

These results revealed a novel function of Nogo-66 in the fate decision of NPCs. This discovery could have profound impact on the understanding of CNS development and could improve the therapy of CNS injuries.

## Introduction

NPCs are a heterogeneous population of mitotically active, self-renewing and multi-potent cells. They have been isolated from the adult and embryonic CNS, as well as the fetal CNS in rodent and human [1], [2]. Transplantation of NPCs has being considered as a potential therapeutic method for CNS injuries. Although NPCs have the potential for neuronal differentiation *in vitro* [3], [4], the majority of NPCs would differentiate into astrocytic phenotype when transplanted into adult non-neurogenic region of CNS such as the spinal cord[5]. Endogenous NPCs have also been observed the predominant glial differentiation after the spinal cord injury [3], [6]. Unlike in the peripheral nervous system, the regeneration ability is extremely limited in adult mammalian CNS because of myelin-associated inhibitors such as Nogo, MAG, and OMgp[7]. Myelin-associated inhibitors are predominantly expressed by oligodentrocytes and they are known to inhibit axon regeneration after injury in adult CNS. Both in vivo and in vitro, the fate decision of NPCs has been suggested to be controlled not only by endogenous genes, such as POU genes, Notch genes, and bHLH genes, but also by exogenous niches. Some outgrowth factors such as EGF, bFGF, LIF and BMPs as exogenous factors could mediate the fate decision of NPCs [8], [9], [10]. Though there is much progress in NPCs differentiation research, the underlying mechanism of NPCs fate decision still remains to be elucidated. In the present study, we have discovered the unknown function of myelin-associated growth inhibitory proteins which promote the glial differentiation and inhibit the neuronal differentiation of NPCs.

## Results

**Figure 1:**
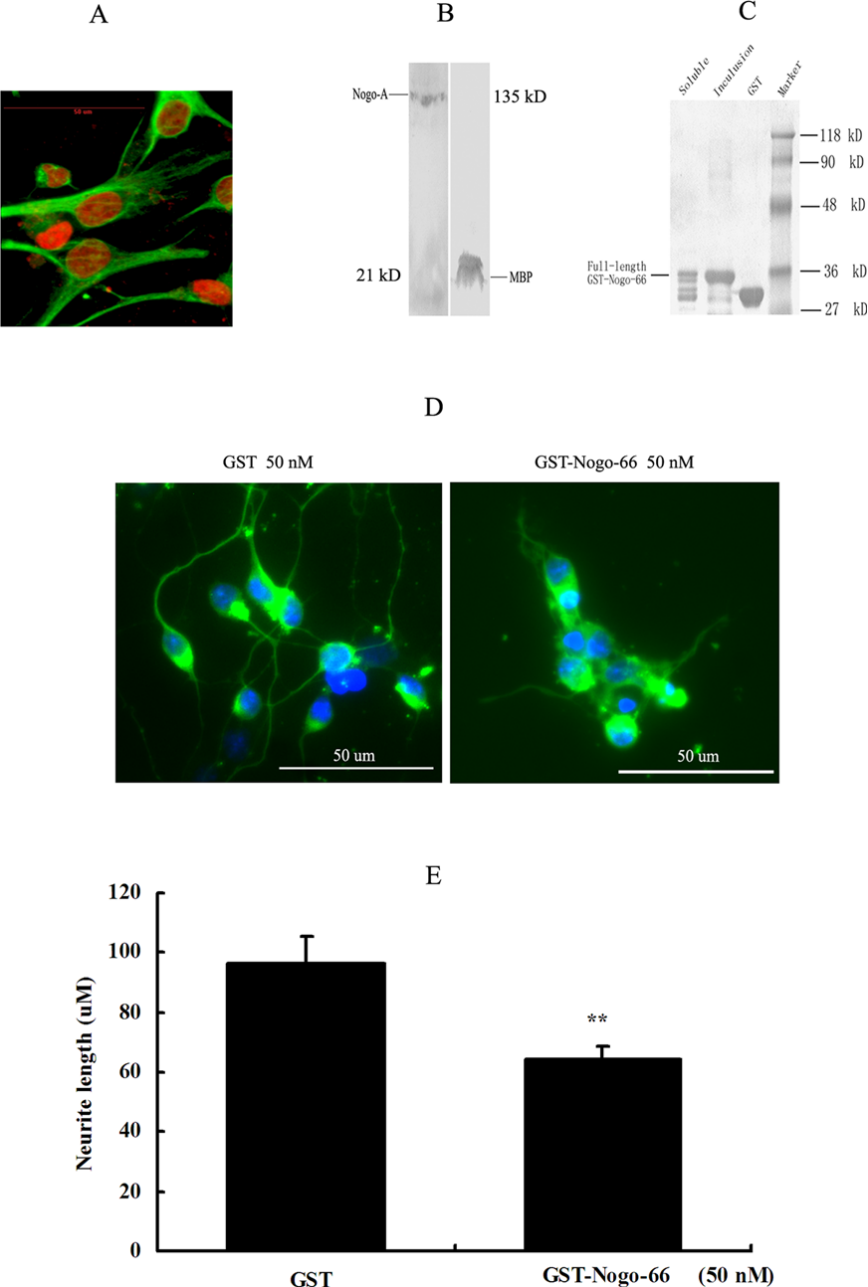
Myelin preparation and GST-Nogo-66 expression. (A) Immunofluorescence analysis of Nestin (green) in the primary culture NPCs. Nuclei were stained by PI (shown in red). Nearly all of the cells were immunostaining positive for Nestin. (B) Western blot analysis detecting Nogo-A and MBP proteins in myelin preparation with a Nogo-A antibody and a MBP antibody. In myelin preparation from adult rat spinal cord not only MBP, a major myelin component, was detected, but Nogo-A was also detected. (C) GST-Nogo-66 SDS-PAGE image. Soluble GST-Nogo-66 purified by glutathione-resin was broken and contained about 30% full-length GST-Nogo-66. Full-length GST-Nogo-66 was about 95% in the inclusion bodies. (D) In vitro analysis of renatured GST-Nogo-66 biological acitivity. Postnatal day 8 rat cerebellar granule neurons were dissociated and placed in culture on slides coated with poly-L-lysine and later supplemented with GST or GST-Nogo-66. After culturing for 48 hours, cells were fixed, permeabalized and stained with a β III tubulin antibody (green) and Nuclei were stained by Hoechst 33342 (blue). Micrographs of the treated cultures showed the inhibitory effects of GST-Nogo-66. (E) Dose-response curve of CGCs treated as in (D) of GST-Nogo-66 protein against neurite length. Results represent the mean neurite length per cell from two independent experiments, with more than 50 cells being measured in three separate wells for each treatment. GST-Nogo-66 significantly inhibited neurite outgrowth at 50 nM concentration (2-tailed *t* test, *P*<0.01).

**Figure 2:**
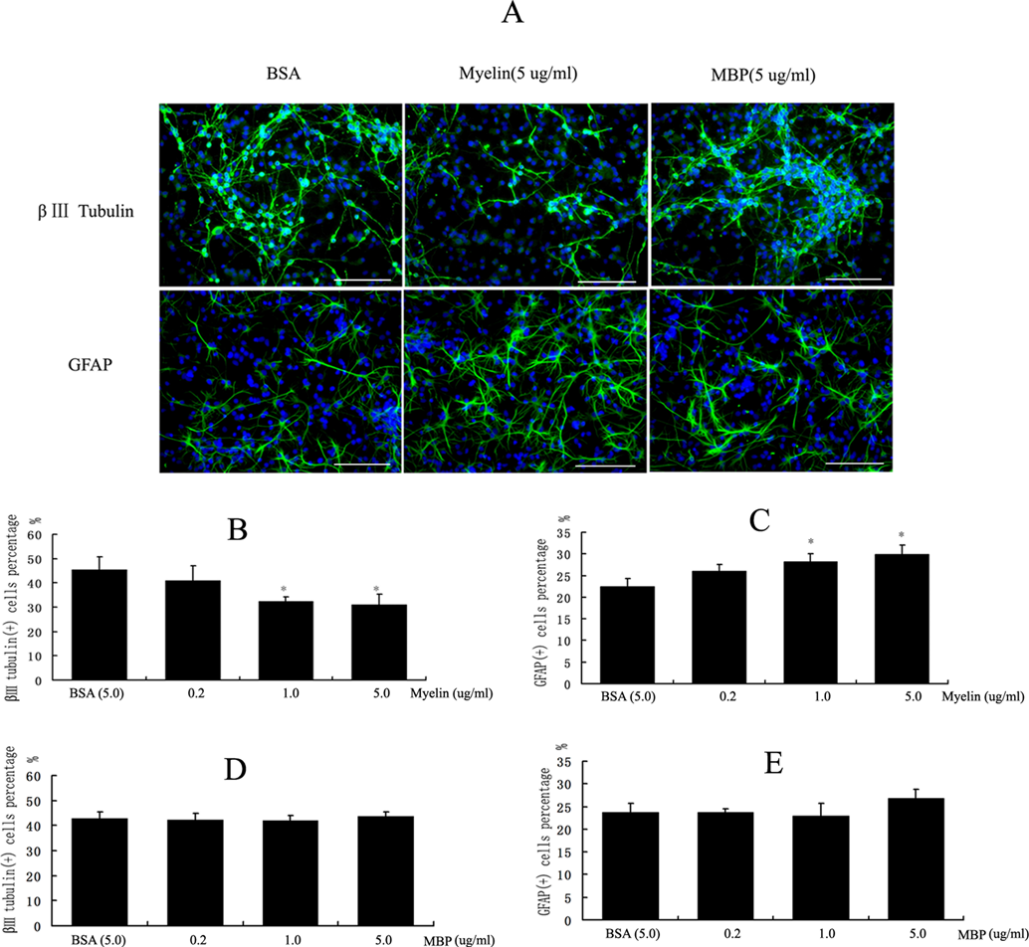
The differentiation of NPCs treated by Myelin and MBP, respectively. (A) The immunostaining images of NPCs treated by myelin and MBP. (B) The result of a β III tubulin antibody immunostaining in NPCs exposure to myelin preparation for 8 days. The proportion of positive cells was 32.35±1.91% and 30.95±4.32% in 1.0 ug/ml and 5.0 ug/ml soluble myelin treatment respectively, significantly lower than 45.38±5.31% in 5.0 ug/ml BSA control (*p*<0.05).(C) The result of GFAP antibody immunostaining in NPCs exposure to myelin preparation for 8 days. The proportion of GFAP positive cells was 28.19±1.1.78% and 29.83±2.16% in 1.0 ug/ml and 5.0 ug/ml soluble myelin treatment respectively, significantly higher than 22.53±1.85% in 5.0 ug/ml BSA control (*p*<0.01).(D, E) Purified MBP did not affect the neural differentiation of NPCs.

We have prepared myelin from adult rat spinal cord and assessed its effect on the differentiation of NPCs (see Fig 1B). After treated with 1.0 or 5.0 ug/ml myelin, NPCs were inhibited to differentiate into neuronal cells (β III tubulin positive cells) and promoted to differentiate into astrocytes (GFAP positive cells) (See Fig 2B and C). There are many components in myelin. We wonder if the major myelin components have the similar effects. Myelin basic protein (MBP) is one of the major myelin proteins, making up 40% of total myelin protein in CNS. We found that MBP protein did not regulate the glial and neuronal differentiation of NPCs (See Fig 2D and E). Some myelin proteins such as Nogo-A, MAG, and OMgp are axon outgrowth inhibitors in CNS. We have hypothesized that these axon inhibitory myelin proteins could mediate the NPCs fate decision. Central to the current understanding of axon inhibitors is the membrane protein Nogo-A. In the myelin preparation, Nogo-A protein was successfully detected by western blot analysis with a Nogo-A antibody (See Fig 1B).

**Figure 3:**
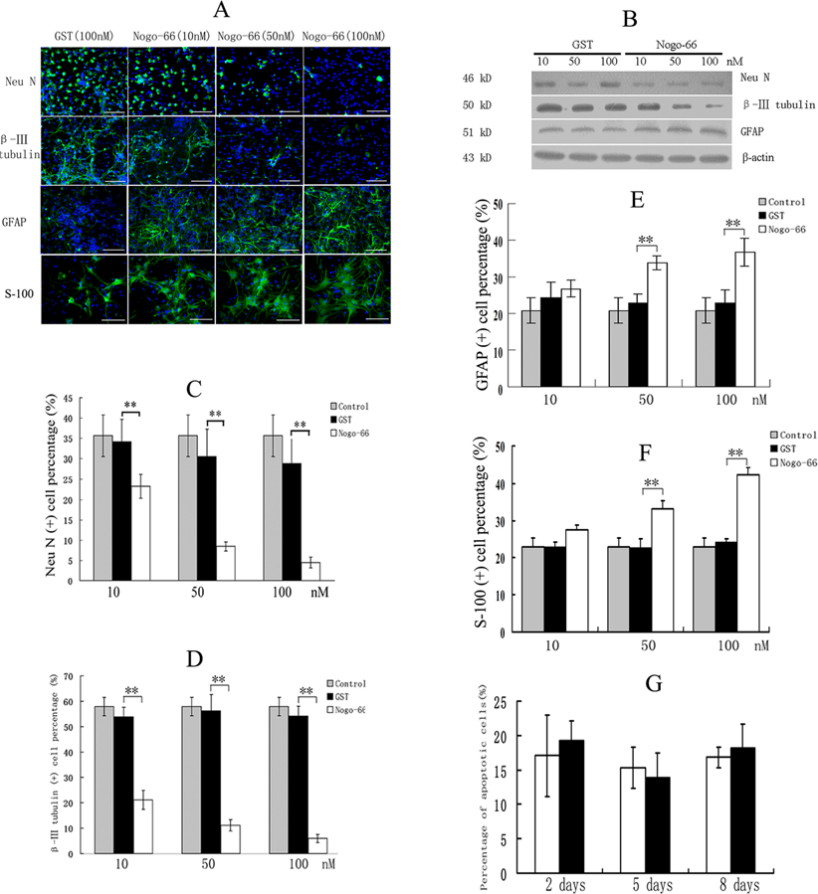
Astroglial induction of NPCs by Nogo-66 for 8 days. (A) Images of immunocytochemistry. (B) In western blot analysis, both NeuN and β III tubulin expressions were decreased and GFAP was upregulated in NPCs induced by Nogo-66. (C, D) The quantitative results of anti-NueN, and β III tubulin antibodies immunocytocheminstry. Nogo-66 significantly inhibited neuronal differentiation in NPCs in dose-dependent manner. (E, F) The quantitative results of anti-GFAP and S-100βantibodies immunocytocheminstry. Nogo-66 significantly induced NPCs differentiation into astroglial cells in dose-dependent manner. (G) Apoptosis assay with Annexin V-Fitc and PI staining by FACS. Nogo-66 did not induce significantly apoptosis compared to GST at day 2, 5, and 8. Data are mean±S.E. Error bars indicate SE. *p<0.05 **p<0.01(n = 3). Bar scale = 100 um.

Nogo-A is predominantly expressed in oligodentrocytes of CNS and its axon growth inhibiting domain of 66 amino acids (Nogo-66) is expressed at the extracellular surface[11] and Nogo-66 receptor (NgR) mediates its regeneration inhibition[12]. The soluble, native GST-Nogo-66 protein purified with a glutathione-resin was broken and only contained about 30% full-length GST-Nogo-66. Thus we have renatured the GST-Nogo-66 from inclusion bodies (see Fig 1C). The renatured GST-Nogo-66 had biological activity and significantly inhibited neurite outgrowth of rat cerebellar granule neurons (See Fig 1D, E). We thus used the renatured Nogo-66 to assess its effect on NPCs differentiation in our study. We observed the differentiation of NPCs treated by Nogo-66 for 8 days *in vitro* (See Fig 3). Nogo-66 promoted NPCs to differentiate into astrogalial cells (GFAP and S-100β positive cells). We found that 50 nM and 100 nM Nogo-66 could significantly increase the proportion of GFAP or S-100β immunostaining positive cells compared to the corresponding dose of GST treatment. It indicated that Nogo-66 could promote astroglial differentiation of NPCs, with the similarity of astrocyte differentiation promotion *in vivo* in previous reports. Meanwhile, both the NeuN and β III tubulin antibodies were used to identify the differentiated neurons in immunostaining analysis and Nogo-66 suppressed the neuronal differentiation of NPCs in a dose-dependent manner. Comparing to β III tubulin expression in neuron cytoplasm and its axons, NeuN was mostly expressed in the nuclei. From the two consistent results, we concluded that Nogo-66 could inhibit the differentiation of NPCs into neurons *in vitro*. And the result was further confirmed by western blot analysis (See Fig 3B). After Nogo-66 treatment, NeuN and β III tubulin expressions were significantly decreased and GFAP expression was significantly increased. In addition, no difference of cell apoptosis percentages between Nogo-66 and GST treatment group was detected by FACS using Annexin V-FITC and propidine iodide (PI) staining (see Fig 3G). The results suggested that the promotion of astroglial differentiation was not the result of the possible enhanced neuronal cell apoptosis induced by Nogo-66. Due to the consistent results with the astrocytic marker, GFAP and S-100βimmunostaining analysis, we considered that Nogo-66 indeed promoted the glial differentiation of NPCs and GFAP was used as the marker of glial cells in our experiments.

**Figure 4:**
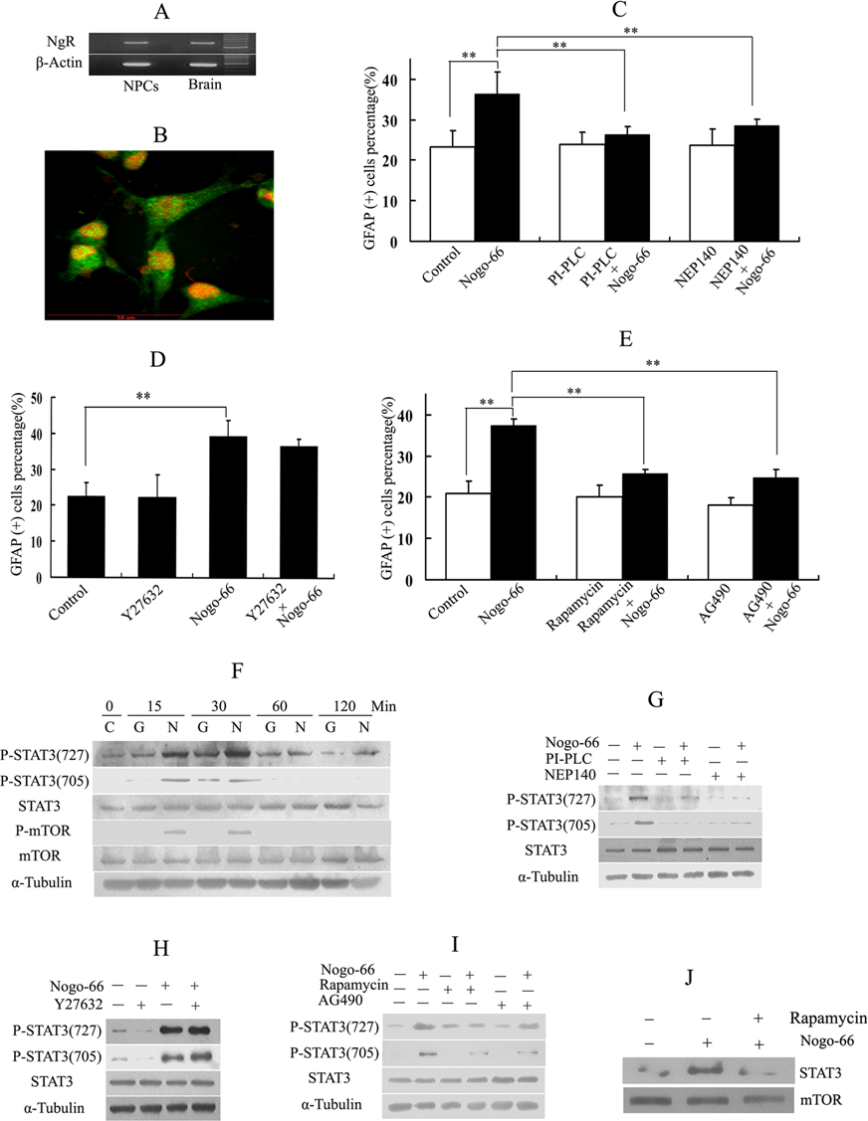
Nogo-66 activated mTOR-STAT3 signaling in NPCs astroglial differentiation. (A) Gel imagine of RT-PCR for NgR mRNA expression in NPCs. (B) Immunocytochemistry for NgR (green) expression in NPCs. Nuclei were stained by PI (shown in red). (C) PI-PLC and NEP1-40 rescued the astroglial induction by Nogo-66. After PI-PLC or NEP1-40 treatment for 2 hours, the proportion of GFAP positive cells induced by Nogo-66 was significantly lower than that induced only by Nogo-66 treatment. (*p*<0.01). (D) The statistical result of GFAP positive cells percentage after Y27632 (RhoA-ROCK inhibitor) treatment. The astroglial induction of Nogo-66 was not rescued by Y27632. (E) Rapamycin and AG490 rescued the astroglial induction by Nogo-66. After rapamycin or AG490 treatment for 2 hours, the proportion of GFAP positive cells induced by Nogo-66 was recovered. (F) Nogo-66 activated the phosphorylation of STAT3 at Ser727 and Tyr705 and phosphorylation of mTOR. After starved for 24 hours in serum-free DMEM medium, total cell lysates from NPCs treated with GST (G) or Nogo-66 (N) (100 nM) for the indicated time were immunoblotted and probed with the indicated antibodies. (G) After starved for 24 hours in serum- free DMEM medium, total cell lysates from NPCs not treated (−) or treated (+) with PI-PLC (1 U/ml) or NEP1-40 for 2 hours and then stimulated with Nogo-66 (100 nM) for 30 minutes were immunoblotted and probed with the indicated antibodies. PI-PLC and NEP1-40 could rescue the phosphorylation of STAT3 activated by Nogo-66. (H) Y27632 did not alter the phosphorylation of STAT3 activated by Nogo0-66. After starved for 24 hours in serum-free DMEM medium, total cell lysates from NPCs not treated (−) or treated (+) with Y27632 (10 uM) for 2 hours and then stimulated with Nogo-66 (100 nM) for 30 minutes were immunoblotted and probed with the indicated antibodies. (I) Rapamycin could inhibit the activated phosphorylation of STAT3 induced by Nogo-66. AG490 strongly inhibited phosphorylation of STAT3 at Tyr705. After starved for 24 hours in serum-free DMEM medium, total cell lysates from NPCs not treated (−) or treated (+) with rapamycin (50 uM) or AG 490 (3ug/ml) for 2 hours and then stimulated with Nogo-66 (100 nM) for 30 minutes were immunoblotted and probed with the indicated antibodies. (J) Rapamycin treatment decreased the complex formation between mTOR and Stat3 in NPCs at the presence of Nogo-66. Nondenatured whole cell lysates were immunoprecipitated with an mTOR antibody before western blot analysis using anti-STAT3 and mTOR, respectively. Data are mean±S.E. Error bars indicate SE. *p<0.05 **p<0.01 (n = 3).

NgR mediates the inhibitory activity of Nogo-66 on axon regeneration in CNS [12]. We tested whether NgR was involved in the glial differentiation of NPCs induced by Nogo-66. To characterize NgR expression in NPCs, we successfully detected the NgR mRNA expression by RT-PCR and protein expression by immunocytochemistry (See Fig 4A and B). NgR is a glycosylphosphatidylinositol (GPI)-linked protein and could be released from membranes by phosphatidylinositol-specific phospholipase C (PI-PLC)[12]. As shown in Figure 4C, after releasing NgR with PI-PLC, the enhanced proportion of GFAP positive cells by Nogo-66 treatment was mostly reversed. To further confirm the above results, another inhibitor NEP1-40 peptide (AD, USA) was used. NEP1-40 was a peptide sequences 1–40 aa of the Nogo-66, acts as the competitive antagonist of NgR and blocks Nogo-66 inhibition of axonal outgrowth in vitro[11]. Similar to the PI-PLC treatment, NEP1-40 mostly rescued the astroglial induction by Nogo-66. Thus we concluded that NgR mediated the astroglial induction of Nogo-66 in NPCs.

It is well known that Nogo-66 inhibits neurite outgrowth and axon regeneration through the RhoA/RHO kinase (ROCK) pathway [13], [14]. Nogo-66 activates RhoA and its downstream target ROCK, and the activity of which can be blocked by the inhibitor Y27632. Y27632 has been used to reduce the inhibitory effect of Nogo-66 on axon regeneration. We tested whether the mechanism of astroglial differentiation promotion by Nogo-66 was the same as that of neurite growth inhibition. Blocking the ROCK activity in NPCs using Y27632 did not affect the astroglial differentiation promotion by Nogo-66(see Fig 4D). Thus the astroglial differentiation promotion of Nogo-66 was not medicated by ROCK pathway.

Janus kinase (JAK)-signal transducers and activators of transcription (STAT3) was involved in the astroglial induction for NPCs or neural stem cells[15], [16], [17]. Interestingly, we have found that Nogo-66 could activate phosphorylation of mammalian target of rapamycin (mTOR) and phosphorylation of STAT3 at Ser727 and Tyr705 (See Fig 4F). We then used mTOR inhibitor rapamcyin and JAK/STAT pathway inhibitor AG490 to test whether mTOR and STAT3 were involved in astroglial induction by Nogo-66 in NPCs. As shown in Figure 4E, both rapamycin and AG490 treatment rescued the enhanced astroglial induction by Nogo-66. In the western blot analysis, we found rapamycin could abolish the activation of phosphorylation of STAT3 at Ser727 and Tyr705 (See Fig 4I). AG490 strongly inhibited the phosphorylation of STAT3 at Tyr705 and weakly inhibit phosphorylation of STAT3 at Ser727. We also found PI-PLC and NEP1-40 peptide treatment could rescue the high level phosphorylation of STAT3 at Ser727 and Tyr705 activated by Nogo-66 (See Fig 4G). But RhoA-ROCK inhibitor Y27632 could not rescue the activated STAT3 phosphorylation by Nogo-66. In addition, we found mTOR and STAT3 could form complex in NPCs and rapamycin treatment decreased the complex formation at the presence of Nogo-66 by immunoprecipitated with anti-mTOR before western blot analysis using a STAT3 antibody (See Fig 4 J).

## Discussion

Many tissues such as muscle, skin, liver, and peripheral nerve tissues have remarkable ability to repair and regenerate after injuries. However, the CNS is very limited to regenerate and this can cause permanent brain damage or paralyses. Recently some inhibitory myelin proteins, such as Nogo-A, MAG, and OMgp in CNS, have been cloned and characterized in neuronal regeneration inhibition. But we know little about these myelin associated regeneration inhibitory proteins in the CNS development and fate decision of NPCs. Although NPCs have the potential for neuronal differentiation *in vitro* [3], [4], the majority of endogenous and exogenous NPCs differentiate into astrocytic phenotype. Because myelin proteins might be involved in forming the niche for NPCs in vivo, they could be involved in mediating the fate decision of NPCs. In this study, we have found that myelin proteins prepared from adult rat spinal cord mediated NPCs fate decision and promoted astroglial differentiation. There are many kinds of components in myelin proteins and MBP was one of the two major myelin proteins. We did not find that MBP had any effect on the differentiation of NPCs. Thus, we hypothesized that the myelin associated inhibitory proteins had the astroglial induction in NPCs differentiation.

Nogo-A, a potent myelin associated inhibitor, is predominantly expressed in oligodentrocytes of CNS and its axon growth inhibiting domain of 66 amino acids (Nogo-66) is expressed at the extra-cellular surface[11]. In the predicted structure of Nogo-A, the N and C-terminus are cytosolic and Nogo-66 residue is in the extracellular or ER lumen localization. But the N terminus of endogenous Nogo-A has been found exposed on the surface of some cells such as fibroblasts, DRG neurons, and myoblasts[18]. Nogo-A could be released during CNS injuries and might be present in the NPCs niche. Thus it may be possible for the interaction between Nogo-66 and NgR to occur in injured CNS and mediate the fate decision of NPCs. In our study, Nogo-66, a potent inhibitory factor in CNS axon regeneration, was found to have a new role in astroglial induction in NPCs. It provided us a novel insight into the function of Nogo-A and other myelin associated factors in the CNS development and regeneration. Nogo-66 receptor (NgR) mediates the regeneration inhibitory signal of Nogo-66 [12]. In this study, we have detected that NgR was expressed in NPCs. After pre-treatment by PI-PLC and NEP140, the astroglial induction of Nogo-66 was partially recovered. Furthermore, we have used PI-PLC, NEP1-40 to recover the activated phosphorylation of STAT3 at Ser727 and Tyr705 by Nogo-66. Thus the results indicated astroglial induction signal of Nogo-66 was mediated by NgR.

The mechanism of Nogo-66 in axon regeneration is well known. After Nogo-66 binding to the NgR, RhoA-ROCK pathway is activated and axon outgrowth is then inhibited. Y27632 is a RhoA-ROCK inhibitor and can partially rescue the regeneration inhibition and promote axon outgrowth of injured neurons both *in vitro* and *in vivo*. After Nogo-66 was found to induce astroglial differentiation of NPCs, we naturally wondered if the RhoA-ROCK pathway was also involved in the astroglial induction. Y27632 treatment did not reverse the astroglial induction of Nogo-66 and failed to rescue the activated phosphorylation of STAT3 induced by Nogo-66. It suggested that the mechanism of its astroglial differentiation induction is not dependent on RhoA-ROCK pathway in NPCs fate decision and different mechanism should be involved.

Extracellular stimulus and the cellular context activate the phosphorylation of STAT3. STAT3 requires phosphorylation on tyrosine (705) and serine (727) residues by independent protein kinase activities for the maximal activation of target gene transcription[19]. Members of the JAK/Tyk family of tyrosine kinases mediate phosphorylation of STAT3 at Tyr705 and mTOR could mediate phosphorylation of STAT3 at Ser727. Activated phosphorylation of STAT3 plays a crucial role in glial differentiation in NPCs or neural stem cells [16], [20]. Some cytokines, such as LIF, BMP2 and CNTF promote glial differentiation of NPCs by activating phosphorylation of STAT3. In the glial differentiation of neural stem cells induced by BMPs, mTOR associates with STAT3 and facilitates STAT activation. In this study, Nogo-66 induced astroglial differentiation of NPCs and activated phosphorylation of STAT3 at both Ser727 and Tyr705 and phosphorylation of mTOR. AG490, a commonly used STAT3 pathway inhibitor[10], could almost entirely rescue the enhanced astroglial induction of Nogo-66 and this indicated that STAT3 and mTOR was indeed involved in the glial induction by Nogo-66.

We have found that rapamycin could mostly abolish the activated phosphorylation of STAT3 at Ser727 and Tyr705. It indicated that mTOR might also mediate the phosphorylation of STAT3 at Tyr705, not only at Ser727. The serine-threonine kinase FKBP12/rapamycin-associated protein (mTOR)/STAT3 pathway played crucial role in glial differentiation induced by BMPs[16], [20] and rapamycin could abolish this effect. Rapamycin can bind to FKBP12 and inhibit the activation of mTOR [21]. With the similar result, we found that rapamycin completely abolished the enhanced astroglial differentiation induced by Nogo-66. In western blot analysis, rapamycin could rescue the activated phosphorylation of STAT3 induced by Nogo-66. In addition, we have found that rapamycin treatment could decrease the complex formation between mTOR and STAT3 by immunoprecipitation with the mTOR antibody before western blot analysis using a STAT3 antibody. These results indicated that mTOR regulated the activation of STAT3 induced by Nogo-66 in NPCs differentiation. The serine-threonine kinase mTOR is an important regulator of cell growth and proliferation by promoting the synthesis of proteins crucial to cell size and cell cycle progression. MTOR can catalyze serine phosphorylation of STAT3 and the activation of STAT3 causes efficient glial differentiation. If rapamycin binds mTOR, the mTOR-STAT3 will be released and Nogo-66 will fail to activate the phosphorylation of STAT3. We thus proposed a model for astroglial induction by Nogo-66 in NPCs fate decision. The binding of Nogo-66 to NgR activated mTOR-STAT3 through a still unclear mechanism. MTOR-STAT3 complex formation is essential for phosphorylation of STAT3 induced by Nogo-66 in NPCs. The activation of STAT3 then induces the glial differentiation.

Myelin associated factors represent an important environmental niche for endogenous and transplanted neural stem cells in CNS, especially at the injured CNS sites. The transplanted neural stem cells or NPCs in the injured CNS mostly differentiated into astroglial lineage. In vitro, Nogo-66 has induced NPCs to differentiate into astroglial cells by activating the mTOR-STAT3 signalling pathway. The glial differentiation promotion by Nogo-66 may contribute to the low efficient neuronal differentiation in endogenous and engrafted neural stem cells in CNS. Our study has provided novel insight into the functions of Nogo-66 and other myelin associated factors in the CNS development and regeneration. Further studies could help to identify important cellular molecules for evolving pharmacological, gene and stem cell therapeutic interventions in CNS regeneration.

## Materials and Methods

### Antibodies and Reagents

Nogo-R (Catalog #: sc-25659, Santa Cruz Biotechnology); GFAP (Catalog #: ab929, ABCAM); NeuN and β III tubulin (Upstate); S-100 beta and α-tubulin (Sigma); P-STAT3 (Ser727), P-STAT3 (Tyr 705) and STAT3 (CST), P-mTOR and mTOR (CST);Nestin (ABCAM) ; Nogo-A (#Nogo-A 12-A, AD);MBP (Boster, China).Myelin basic protein (MBP), bovine puried (Upstate). Rapamycin (Sigma); AG490 (Biosource); Y27632 (Sigma); PI-PLC (Sigma); NEP1-40 (Calbiochem).

### Cell Culture and Treatments

NPCs were prepared as described previously [22] with slightly modification. The telencephalon of neonatal Sprague Dawley (SD) rats were carefully dissected in serum-free DMEM/F12 medium and triturated. The cell suspension was cultured in 25 cm^2^ tissue culture flask (Corning) in serum-free DMEM/F12 medium with B27 supplement, 10 ng/ml bFGF(PeproTech EC), and 20 ng/ml EGF(PeproTech EC). After 8-day culture, neurosphere were formed and the cells were enzymatically dissociated for following experiments.

For Immunocytochemistry, NPCs were plated in 48 well culture plate (Corning) at 10^4^ cells per well. The maintaining medium was serum-free DMEM (high glucose) with N2 supplement. In the presence of myelin, MBP, GST, or Nogo-66 for 8-day, the terminal differentiated cells was assessed by immunocytochemistry. For the biochemical assays, cells were treated with factors and harvested at the time points mentioned in the figure legends.

### Myelin Preparation

Myelin preparation from adult rat spinal cord was produced according to previous report[23]. To isolate myelin, tissue is homogenized in 0.3 M sucrose and layered on a gradient of 1.23 and 0.85 M sucrose. Samples are centrifuged for 45 min at 75 000 g and the crude myelin fraction is collected at the 0.85/1.23 M interface. Crude myelin is washed twice by osmotic shock, resuspended in 0.32 M sucrose, layered over 0.85 M sucrose, centrifuged, and collected from the 0.32/0.85 M interfaces. After removal of excess sucrose myelin is resuspended 1∶1 in DMEM, homogenized and stored at −80°C until use.

### Apoptosis Assay

Cell apoptosis was measured with FACS. For each sample, approximately 1×10^6^ cells were washed twice with PBS and then resuspended in 200 µL of binding buffer (10 mM HEPES, 140 mM NaCl, 5 mM CaCl_2_). 10 µL of Annexin V-FITC stock solution (20 µg/mL) was added to the cells and incubated for 30 min at 4°C. The cells were then further incubated with 5 µL propidine iodide (PI, 50 µg/mL) and were immediately analyzed on a FACSC-LSR (Becton-Dickton) equipped with Celluest (Becton-Dickton) software.

### GST-Nogo-66 Protein Expression

Nogo-66, a portion of Nogo-A mRNA encoding the 66-residue lumenal/extracellular fragent was ampilified and ligated to pGEX-4T-1 plasmid (Amersham Biosciences) to yield a prokaryotic expression vector for the GST-Nogo-66 protein. *E.coli* transformed with the plasmid was induced with 0.1 mM IPTG. Soluble, native GST-Nogo-66 protein purified using a glutathione-resin was broken and only contained about 30% full-length GST-Nogo-66. Most of the GST-Nogo-66 proteins were in inclusion bodies and were full-length (see Fig 1). GST-Nogo-66 from inclusion bodies was renatured by dilution renature method. Briefly, the recombinant protein was isolated from Escherichia coli as inclusion bodies by sonication and centrifugation, and then dissolved with 8 M urea and renatured by dilution using renature buffer (0.5 M NaCl, 5 mM GSH, 1 mM GSSH, 50 mM, 50 mM, 1 mM EDTA-Na2). The biological activity of the GST-Nogo-66 was tested according to previous report[24]. Postnatal 8 days rat cerebellar granule neurons (CGCs) were dissociated and placed in culture on slides coated with poly-L-lysine with DMEM/F12 containing 10% FBS for 30 min and then supplemented with control GST protein, or the inhibitory proteins GST-Nogo-66 in DMEM/F2 medium plus N2 supplement . After growth for 48 hours, cells were fixed, permeabilized and stained with a beta-3 tubulin antibody. Micrographs of the treated cultures show the inhibitory effects of GST-Nogo-66. In this study, the renatured GST-Nogo-66 with biological activity was used.

### Immunoblotting and Immuoprecipitation

Cell lysates were subjected to 8% SDS–PAGE and transferred to nitrocellulose membranes. For immunoprecipitation, 500 ul (500 ug) cell lysates were incubated with anti-mTOR antibody (1∶100) overnight at 4°C. After incubation with protein A–Sepharose (1∶1 vol/vol), the immune complexes were washed twice with PBS and heated to 70C in SDS-PAGE loading buffer. The blots were probed with indicated primary antibodies, followed by secondary antibodies conjugated with HRP. Fluorescent signals were detected with ECL system (Pierce). For immunoblotting, primary antibodies were diluted as follow: NeuN, β III tubulin, α-tubulin, P-STAT3 (Ser727), P-STAT3 (Tyr 705), STAT3 (CST), P-mTOR and mTOR (CST) at 1∶1000; Nogo-A at 1∶300, MBP at 1∶200, GFAP 1∶100.

### Immunocytochemistry

NPCs were plated in 48 well culture plate (Corning) at 10^4^ cells per well. After 8-day GST or GST-Nogo-66 administration, cells were fixed by 4% formaldehydum polymerisatum (Merck) for immunocytochemistry. Immunocytochemistry was performed using theβ III tubulin, neuronal nuclei (NeuN), and GFAP antibody, respectively. The primary antibodies were incubated overnight at 4°C and then incubated with the secondary FITC-conjugated antibodies and Hoechst33342 (1 µg/ml) for 1 hr at room temperature. Primary antibodies were diluted as follow: NeuN and β III tubulin at 1∶800; NgR at 1∶400, GFAP at 1∶6; and Nestin at 1∶600. No immune IgG was used as control and did not find nonspecific staining. After immunostaining of differentiated cells, images of Hoechsst dye staining neucleus DNA (to identify the total number of cells in the field) and the respective antigens were captured with a Zeiss Axiovert 200 (Carl Zeiss). The respective images were overlaid and the percentage of antigen positive cells was calculated. Six random fields/well from three replicated wells were counted.

### RT-PCR

Total RNA was extracted using TRIzol reagent (Invitrogen life technologies) according to the manufacturer's protocol. Primers were designed as following: NgR sense 5′ GGGCAACCTCACGCGCATCT 3′, NgR anti-sense 5′ CGGGCAAAGTCCCAAAT 3′; β-actin sense 5′ GTCCCTGTATGCCTCTGGTC 3′, β-actin anti-sense 5′ GGTCTTTACGGATGTCAACG 3′. Primers for β-actin gene were cycled 24 times and those for others were cycled 28 times.

### Statistical analysis

Data were mean±S.E. Statistical analyses in this study were performed with ANOVA. When significant differences were found, post-hoc comparisons were made using the Tukey honestly significant difference test. *P<0.05 was considered significant in all instances.
